# Aldehyde Dehydrogenase 2 Protects the Kidney from Ischemia–Reperfusion Injury by Suppressing the I*κ*B*α*/NF-*κ*B/IL-17C Pathway

**DOI:** 10.1155/2023/2264030

**Published:** 2023-02-21

**Authors:** Yiwen Chen, Yan Xiong, Jun Luo, Qianchao Hu, Jianan Lan, Yongkang Zou, Qin Ma, Hanlin Yao, Zhongzhong Liu, Zibiao Zhong, Qifa Ye

**Affiliations:** ^1^National Quality Control Center for Donated Organ Procurement, Hubei Key Laboratory of Medical Technology on Transplantation, Hubei Clinical Research Center for Natural Polymer Biological Liver, Hubei Engineering Center of Natural Polymer-Based Medical Materials, Zhongnan Hospital of Wuhan University, Institute of Hepatobiliary Diseases of Wuhan University, Transplant Center of Wuhan University, Wuhan, Hubei 430071, China; ^2^Research Center of National Health Ministry on Transplantation Medicine Engineering and Technology, The 3rd Xiangya Hospital of Central South University, Changsha 410013, China

## Abstract

**Objective:**

Ischemia–reperfusion injury (IRI) is an important cause of delayed functional recovery after transplantation. This study is aimed at investigating the molecular mechanism of ALDH2 in a kidney ischemia–reperfusion model based on RNA-seq.

**Methods:**

We performed kidney ischemia–reperfusion in ALDH2^−/−^ and WT mice and evaluated kidney function and morphology using SCr, HE staining, TUNEL staining, and TEM. We used RNA-seq to compare mRNA expression in ALDH2^−/−^ and WT mice after IR, and then, we verified the related molecular pathways by PCR and western blotting. In addition, activators and inhibitors of ALDH2 were used to alter the activity of ALDH2. Finally, we established a model of hypoxia and reoxygenation in HK-2 cells and clarified the role of ALDH2 in IR by interfering with ALDH2 and using an NF-*κ*B inhibitor.

**Results:**

After kidney ischemia–reperfusion, the SCr value increased significantly, kidney tubular epithelial cells were damaged, and the apoptosis rate increased. In the microstructure, mitochondria were swollen and deformed, and ALDH2 deficiency aggravated these changes. The NF-*κ*B pathway and IL-17 pathway were significantly enriched in ALDH2^−/−^ mice compared with WT mice according to KEGG enrichment analysis of the RNA-seq data. The PCR results showed that the mRNA expression levels of I*κ*B*α* and IL-17B, C, D, E, and F were significantly higher than those in the WT-IR group. Western blot verification results showed that ALHD2 knockdown resulted in increased phosphorylation of I*κ*B*α*, increased phosphorylation of NF-*κ*B, and increased expression of IL-17C. When we used ALDH2 agonists, the number of lesions and the expression levels of the corresponding proteins were reduced. Knockdown of ALDH2 in HK-2 cells resulted in a higher proportion of apoptotic cells after hypoxia and reoxygenation, but inhibiting the phosphorylation of NF-*κ*B prevented the increase in apoptosis and reduced the protein expression level of IL-17C.

**Conclusion:**

ALDH2 deficiency can lead to the aggravation of kidney ischemia–reperfusion injury. RNA-seq analysis and validation by PCR and western blotting revealed that this effect may be due to the promotion of I*κ*B*α*/NF-*κ*B p65 phosphorylation during ischemia–reperfusion caused by ALDH2 deficiency, which then leads to an increase in inflammatory factors, including IL-17C. Thus, cell death is promoted, and kidney IRI is eventually aggravated. We link ALDH2 deficiency with inflammation, revealing a new idea for ALDH2-related research.

## 1. Introduction

Kidney transplantation is the only treatment for end-stage kidney disease [1]. However, during kidney transplantation, the kidney undergoes thermal ischemia, cold ischemia, and ischemia–reperfusion, which inevitably leads to ischemia–reperfusion injury (IRI) [2, 3], resulting in delayed graft function (DGF) and reducing the long-term graft survival rate [3]. Energy depletion during ischemic lysosomal membrane rupture inhibits the cytoplasmic activity of Na^+^/K^+^ ATPase and calcium overload [4, 5], and reperfusion further induces reactive oxygen species (ROS) generation and the activation of intracellular calcium-dependent proteolytic enzymes [6], resulting in further damage, activation of cell death, and aseptic inflammation [7].

Aldehyde dehydrogenase 2 (ALDH2) is an acetaldehyde dehydrogenase and is made up of 517 amino acids and 4 subunits of an autotetraploid stable protein structure [8]. A large number of important organs, such as the brain, heart, liver, kidneys, and other tissues, express ALDH2 in the mitochondria [9]. The ALDH2 variant ALDH2^∗^504Lys exists only in the East Asian population, with a frequency as high as 40% in some collected samples [10]. The enzymatic activity of this variant is far lower than the normal level, and these individuals have a higher risk of cancer, Alzheimer's disease, and other diseases than normal individuals [11]. Further research is needed to understand the mechanism of ALDH2 deficiency in IRI. Studies have shown that ALDH2 overexpression can reduce the synthesis of toxic aldehydes during IR, thus protecting mitochondrial function during IR and alleviating IRI [12]. It has been proved that Alda-1, an agonist of ALDH2, can be used to alleviate ischemia–reperfusion injury of the brain, heart, liver, and other organs in animals.

Previous studies in our laboratory showed that the expression of ALDH2 was increased in rabbit kidneys during mechanical perfusion at hypothermic temperature, which alleviated apoptosis after IR by affecting the AMPK pathway and that ALDH2 played a positive role in autophagy through the Akt-mTOR pathway [13–15]. Thus, we established ALDH2-knockout (ALDH2^−/−^) mice to investigate the role of ALDH2 in kidney protection.

Nuclear factor *κ*B (NF-*κ*B) is a transcription factor found in all mammals. Together with AKT, mTOR, and other molecules, it regulates several metabolic processes, including inflammation, apoptosis, cell survival, and autophagy [16–18]. NF-*κ*B plays a crucial role in IR-induced acute kidney injury (AKI) and mediates inflammatory responses [19–21]. During IR, I*κ*B*α*, an upstream inhibitor of NF-*κ*B, is phosphorylated and degraded, resulting in the activation of the p65 and p50 subunits of NF-*κ*B, which are transferred from the cytoplasm to the nucleus [22]. The activation of relevant inflammatory factor transcription, the induction of aseptic inflammation after ischemia–reperfusion (IR), and how to inhibit the activation of NF-*κ*B safely and effectively are current research directions in IR. In this study, we found changes in the mRNA expression of many molecules in the NF-*κ*B pathway in an IRI model after ALDH2^−/−^. Therefore, the lack of ALDH2 in IR may cause the upregulation of the NF-*κ*B pathway, and the specific mechanism needs to be further studied.

To better understand how ALDH2 alleviates kidney injury in kidney ischemia–reperfusion injury, we constructed ALDH2 knockout (ALDH2^−/−^) mice using a model of severe bilateral ischemia–reperfusion (IR) injury [23]. This method aggravated the damage while ensuring the short-term survival of the mice. We found that ALDH2^−/−^ mice had significantly higher degrees of AKI, inflammation, and general tissue damage. ALDH2 deficiency increases the expression of I*κ*B*α* and stimulates the phosphorylation of I*κ*B*α*/p65, which, in turn, causes an increase in IL-17. Inhibition of I*κ*B*α*/p65 phosphorylation and IL-17C expression plays key roles in ALDH2-mediated protection for renal IRI.

## 2. Materials and Methods

### 2.1. Animals

Male 8-week-old C57BL/6 mice were obtained from Beijing Weitonglihua Experimental Animal Technology Co., Ltd. ALDH2 knockout male mouse (ALDH2^−/−^) was provided by Wuhan Xian Ran Biological Co., Ltd. Mice were reared in the Animal Experimental Center at Zhongnan Hospital of Wuhan University for 12 hours in light/dark mode, with plenty of food and water.

### 2.2. Renal IRI Model

The mice were fasted for 12 hours before operation and could not restrain water. The mice were anesthetized with 50 mg/kg pentobarbital sodium injected intraperitoneally. The body temperature was maintained at 37°C and monitored intraoperatively using a temperature controller with a heating pad (JR-1/2, Taimeng, Chengdu, China). Then, through a midline abdominal incision, a nontraumatic vascular clamp (Roboz, USA) was used to clamp the bilateral renal pedicles for 45 minutes to induce ischemia, which was opened to confirm renal reperfusion and the abdomen was closed. After the operation, the mice were fed with a normal diet, and each mouse was supplemented with an appropriate amount of sterile normal saline and a small amount of meloxicam for analgesia. 24 h after reperfusion, the mice were sacrificed for excessive pentobarbital sodium and cervical dislocation, and renal and blood samples were collected for further analysis.

### 2.3. Biochemical Analysis

The collected blood samples were centrifuged at 3500 rpm for 10 min, and the serum levels of Cr and BUN were measured by automatic analyzer of clinical samples in Zhongnan Hospital of Wuhan University.

### 2.4. H&E Staining

H&E was performed on all renal samples after sectionalization, paraformaldehyde fixing for 24 hours, dehydration by ethanol, and embedding in paraffin. The pathological morphological changes of renal tissues were observed under a light microscope, and Paller scores [24] were performed by renal pathologists who were unaware of the experiment under a 400x light microscope. Ten high-magnification visual fields were randomly selected, and 10 renal tubules were selected for each visual field for evaluation. The criteria were as follows: 1 point for morphological change of renal tubular epithelial cells, 1 point for damage of brush edge, 2 points for shedding brush edge, 2 points for appearance of tubular type, 1 point for shedding necrotic cells in the lumen, and 1 point for obvious deformation and broadening of tubular diameter. The higher the score is, the more severe the renal tubular injury is.

### 2.5. TdT-Mediated dUTP Nick-End Labeling

Follow the manufacturer's instructions when staining fresh frozen tissue sections. TUNEL staining was performed using in OneStep TUNEL Apoptosis Assay Kit (Beyotime, China). And the nucleus staining was used 2-(4-amidinophenyl)-6-indolecarbamidine dihydrochloride (DAPI) (Beyotime, China). TUNEL-positive cells in kidney tissue were counted and analyzed in random field of vision of the renal cortex (×200 magnification).

### 2.6. RNA Sequencing

Total mRNA was extracted from renal tissues of WT-IR and KO-IR groups (*n* = 3) using TRIzol. The RNA quality was verified using a 2100 Bioanalyzer and ND-2000, and the samples were sequenced by Illumina HiSEQ 4000 of Shanghai Magi Biomedical Technology Co., Ltd. All differentially expressed genes were used for heat maps analysis and KEGG enrichment analysis. For KEGG enrichment analysis, *P* < 0.05 was considered statistically significant.

### 2.7. TEM Assay

The renal tissue fixed with 2.5% glutaraldehyde was made into nanoscale sections and observed under an electron microscope. Single-cell morphology was observed at 1500x, and the nucleus and mitochondria were observed at 6000x [25].

### 2.8. Western Blot Analysis

Complete lysates were extracted from the renal for western blotting. A phosphatase inhibitor was also added to the lysate. The membrane was incubated with the corresponding primary antibody (1 : 800) at 4°C for 12 h. Primary antibodies used in these experiments were as follows: I*κ*B*α*, phospho-I*κ*B*α*, NF-*κ*B p65, phospho-NF-*κ*B p65, IL-17C, and *β*-actin (ABclonal, China). The proteins were incubated and imprinted with enhanced chemiluminescence (ECL) reagent (Biosharp, China), and the protein expression was quantified by optical density analysis using ImageJ software. All bands were tested for *β*-actin (1 : 3000, ABclonal, China) content as a standard control for sample loading.

### 2.9. RT-qPCR

TRIzol (Yisheng, China) was used to isolate total RNA from mouse renal samples and HK-2 cells. According to the kit instructions, the excess DNA was removed and reverse transcribed into cDNA. The expression of target gene was detected by SYBR Green quantitative real-time polymerase chain reaction. With GAPDH as internal reference, the changes of RNA level were calculated by 2^-*ΔΔ*Ct^ method and analyzed by the Student *T*-test. The primers used are presented in Table S1.

### 2.10. Analysis of ALDH Enzyme Activity

According to the manufacturer's instructions, ALDH2 activity was detected using an ALDH assay kit (Solarbio, China). ALDH was extracted from renal tissue using the kit, and then, the activity of ALDH was assessed by measuring NAD+ production at 340 nm using an experimental microplate reader (Thermo, Beijing, China).

### 2.11. Cell Culture

HK-2 cells purchased from the Biological Sample Bank of Wuhan University were cultured at 37°C in humidified air (5% CO_2_) using F12/MEM (Servicebio, China) medium containing 10% fetal bovine serum (Gibco, South America) and 1% penicillin-streptomycin (10, 000 U/mL, Servicebio, China). The medium was changed daily. Each time about 80% cell density is passed.

### 2.12. Construction of Stable Cell Lines

The virus loaded with interfering plasmids was purchased from Shanghai Genechem Co., Ltd. The virus was added into HK-2 cell culture medium for 16 h and replaced with normal culture medium. After 72 h, the HK-2 cells were added into puromycin culture medium for 2 weeks. The siRNA sense strand sequences were as follows: ALDH2: 5′-GCAGGCATACACTGAAGTGAA-3′ and 5′-TTCACTTCAGTGTATGCCTGC-3′. Finally, we obtained stable cell lines with low expression of ALDH2.

### 2.13. Oxygen and Glucose Deprivation and Reoxygenation

Cells were cultured in a three-gas incubator (1% O_2_) for 24 h with DMEM (Servicebio, China) free of glucose and then in normoxic complete F12/MEM medium for 6 h.

### 2.14. Statistical Analysis

SPSS24.0 software was used for statistical analysis, and GraphPad Prism 8.0.1 was used for plotting. The measurement data in accordance with normal distribution were expressed by *x* ± *s*, one-way ANOVA was used between multiple groups, and independent sample *T*-test was used between pairs. *P* < 0.05 was considered statistically significant.

## 3. Results

### 3.1. ALDH2 Deficiency Exacerbates Kidney IRI

**Figure 1:**
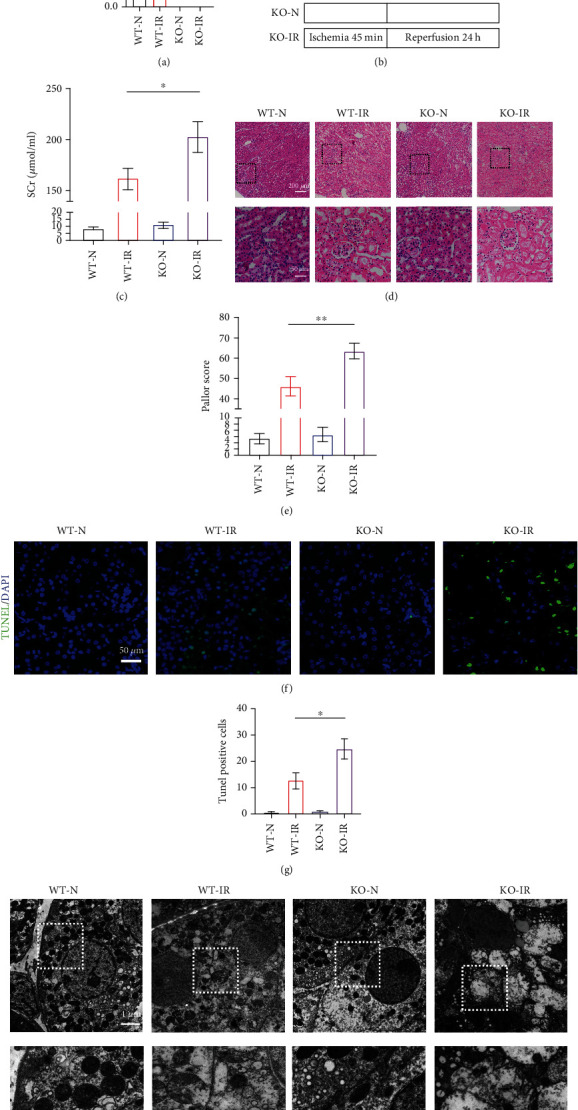
ALDH2 deficiency exacerbates renal ischemia–reperfusion injury. WT mice and ALDH2^−/−^ mice were subjected to either sham operation or kidney ischemia for 45 minutes and reperfusion for 24 hours. (a) Kidney lysates from WT mice and ALDH2^−/−^ mice were subjected to western blot analysis for ALDH2. *n* = 3/group. (b) An illustration of the injury model used in this study. (c) Serum creatinine (SCr) were measured after 24 h of reperfusion. *n* = 6/group. (d) H&E staining showed renal tubule epithelial cell injury and red blood cell deposition. Scale bar: 200 *μ*m, 50 *μ*m. (e) Paller score was performed using a semiquantitative damage assessment of renal tubular epithelial cells for each sample. *n* = 6/group. (f) Representative images of TUNEL staining (green) and DAPI (blue). Scale bar: 100 *μ*m. (g) The percentage of TUNEL staining-positive cells in the total cells in the random field. *n* = 3/group. (h) Morphology of renal tubular epithelial cell was observed under TEM. *n* = 3/group. Scale bar: 20 *μ*m, 10 *μ*m. Data are presented as the mean ± SD. ^∗^*P* < 0.05, ^∗∗^*P* < 0.01, and ^∗∗∗^*P* < 0.001 by *t*-test.

To explore the role of ALDH2 in mouse kidneys, ALDH2 knockout mice were constructed, and the expression of ALDH2 was verified (Figure 1(a)). A bilateral kidney IRI model (Figure 1(b)) was established in mice, and we collected kidney tissue and blood samples for further testing. Creatinine (SCr) levels in ALDH2^−/−^ mice were significantly higher than those in the WT group (Figure 1(c)). The kidneys of ALDH2^−/−^ mice showed more severe damage than those of WT mice (Figures 1(d) and 1(e)). Consistent with these data, ALDH2^−/−^ mice had more TUNEL-positive cells in their kidneys after ischemia–reperfusion than WT mice (Figures 1(f) and 1(g)). The TEM results showed that the effects on ALDH2^−/−^ ischemic kidneys, with mitochondrial swelling and even rupture, disappearance of mitochondrial structure, and nuclear pyknosis, were more serious than those on WT kidneys (Figure 1(h)). These results suggest that ALDH2 knockout exacerbates IR-induced kidney injury.

### 3.2. The RNA-seq Results Showed That the NF-*κ*B and IL-17 Pathways Were Enriched during IR after ALDH2 KO

**Figure 2:**
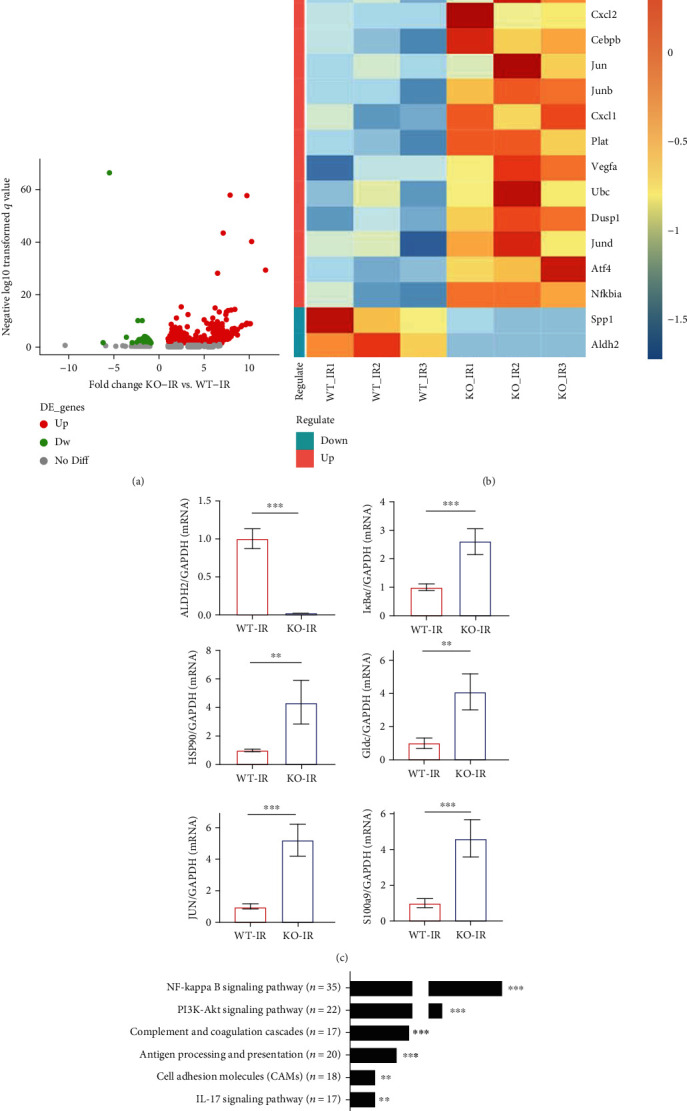
RNA-seq results showed that ALDH2 deficiency led to changes in inflammatory molecular pathways. (a) The volcano plots showed the differential genes between the KO-IR and WT-IR groups. (b) Heat map showing mRNA differences in RNA-seq. (c) Expression of ALDH2, I*κ*B*α*, HSP90, Gldc, JUN, and S100a9 mRNA was determined by RT-qPCR. GAPDH was the standard. *n* = 5/group. Data are presented as the mean ± SD. ^∗^*P* < 0.05, ^∗∗^*P* < 0.01, and ^∗∗∗^*P* < 0.001 by *t*-test. (d) KEGG enrichment analysis revealed the signal pathway of the concentrated expression of differential genes in the KO-IR and WT-IR groups.

To examine ALDH2-dependent global changes in gene expression in ALDH2^−/−^ and WT mice, we performed RNA-Seq. Compared with WT mice after IRI, 140 genes were upregulated and 11 genes were downregulated in ALDH2^−/−^ mice (*P* value < 0.05 and abs [log fold change (logFC)] > 1.5) (Figure 2(a)). In Figure 2(b), differential mRNA expression is displayed as a hierarchical clustering map. Upregulation of various genes of interest, including HSP90, Gldc, S100a9, I*κ*B*α*, and JUN, was confirmed by real-time qPCR (RT-qPCR) (Figure 2(c)). Comparing ALDH2^−/−^ mice with WT mice, KEGG analysis revealed differential expression of genes involved in inflammatory pathways (Figure 2(d)).

**Figure 3:**
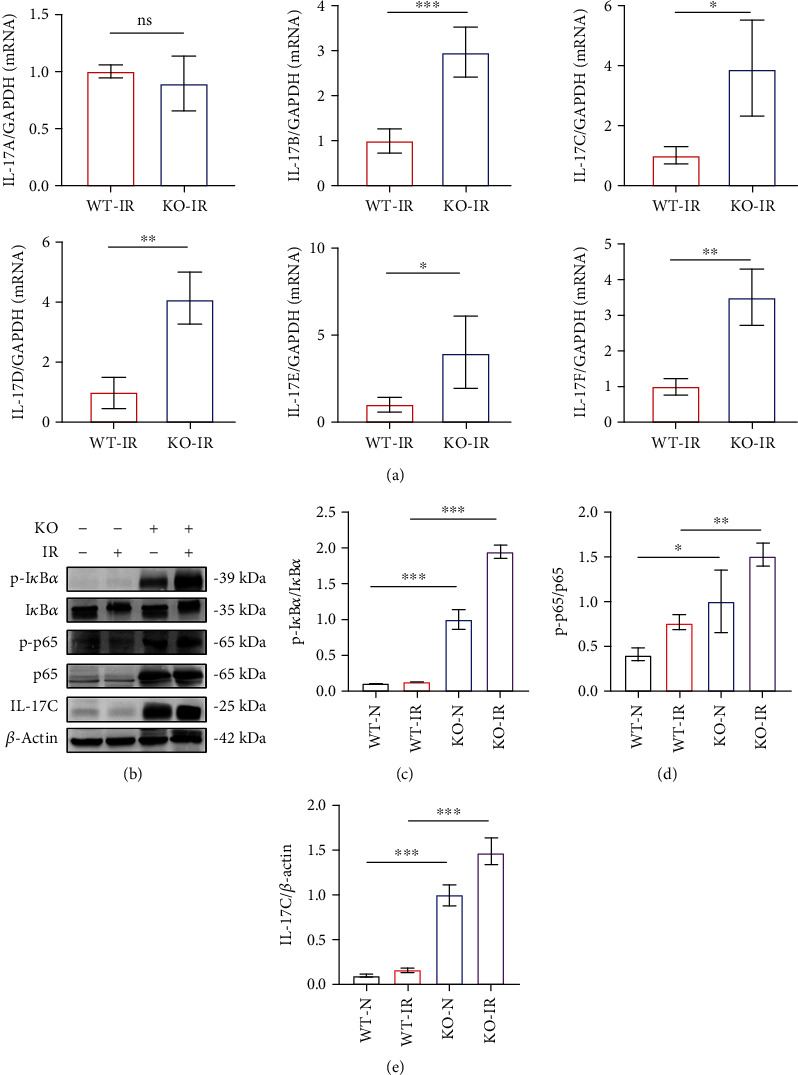
ALDH2 deficiency resulted in increased phosphorylation of I*κ*B*α*/NF-*κ*B P65 and il-17C expression. (a) The expression of IL-17 family mRNA in the kidney was determined by real-time PCR. GAPDH was the standard. *n* = 5/group. (b) The western blot band of p-I*κ*B*α*, I*κ*B*α*, p-p65, p65, and IL-17C in kidney lysates. *β*-Actin was the standard. *n* = 3/group. (c)–(e) Western blot analysis for p-I*κ*B*α*, I*κ*B*α*, p-p65, p65, and IL-17C protein in kidney samples from each group. *n* = 3/group. Data are presented as the mean ± SD. n.s.: not significant. ^∗^*P* < 0.05, ^∗∗^*P* < 0.01, and ^∗∗∗^*P* < 0.001 by *t*-test.

The sequencing results showed high expression of many inflammatory chemokines and related genes, such as CXCL1, CXCL2, S100a8, S100a9, and MMP9. ALDH2^−/−^ may cause a worsened inflammatory response, which seems to be consistent with the pathological manifestations in the KO-IR group. We hypothesized that ALDH2 KO could affect the NF-*κ*B pathway and even the IL-17C pathway to aggravate IRI in the kidney. We performed RT-qPCR to analyze six members of the IL-17 family and found that the expression levels of IL-17B, IL-17C, IL-17D, and IL-17F were significantly altered (Figure 3(a)), so these factors may be associated with ALDH2. Studies have reported that IL-17A and IL-17C expressed in the kidney can affect the level of inflammation in the kidney [26]. Therefore, we focused on exploring the relationship between ALDH2 and IL-17C.

### 3.3. ALDH2 Deficiency Leads to the Initiation of Downstream Inflammatory Pathways by Activating I*κ*B*α*/p65 Phosphorylation

**Figure 4:**
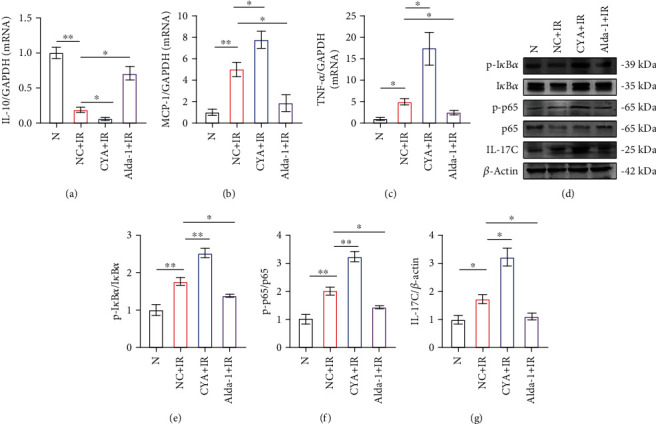
Activation of ALDH2 reduced inflammation and inhibited the phosphorylation of I*κ*B*α*/NF-*κ*B p65 in I/R. (a)–(c) The expression of IL-10*β*, MCP-1, and TNF-*α* mRNA in the kidney was determined by real-time PCR. GAPDH was the standard. *n* = 3/group. (d) The western blot band of p-I*κ*B*α*, I*κ*B*α*, p-p65, p65, and IL-17C in kidney lysates. *β*-Actin was the standard. *n* = 3/group. (e)–(g) The western blot analysis of p-I*κ*B*α*, I*κ*B*α*, p-p65, p65, and IL-17C protein in kidney samples from each group. *n* = 3/group. Data are presented as the mean ± SD. ^∗^*P* < 0.05, ^∗∗^*P* < 0.01, and ^∗∗∗^*P* < 0.001 by *t*-test.

Based on PCR results, I*κ*B*α* mRNA expression was elevated, but in the reported during kidney ischemia–reperfusion, I*κ*B*α* was phosphorylated to activate the phosphorylation of NF-*κ*B P65 and was then degraded. A protein imprinting assay was performed (Figures 3(b)–3(e)), and high phosphorylation of I*κ*B*α* and P65 was observed in the KO group regardless of IR status, and IL-17C was also highly expressed in the KO group. After IR, the phosphorylation levels of I*κ*B*α* and p65 further increased, and the expression level of IL-17C increased. Due to the high expression of proteins in the KO group, it is difficult to observe how the expression levels of these proteins change in the IR model of WT mice. Here, we imprinted the proteins of the N and IR groups of WT mice separately and found that the phosphorylation levels of I*κ*B*α*, p65, and IL-17C increased in WT mice after IRI (Figures 4(d)–4(g)), as reported in the literature [27].

### 3.4. Activation of ALDH2 Alleviates Kidney IRI in WT Mice

**Figure 5:**
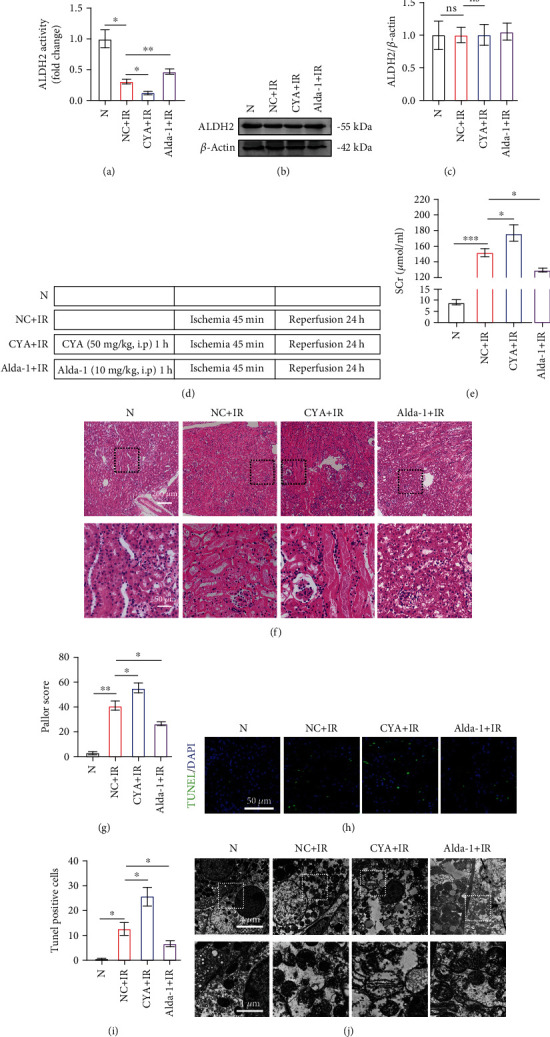
Activation of ALDH2 alleviates kidney ischemia–reperfusion injury, while inhibition aggravates the injury. WT mice were pretreated with solvent, CYA, and Alda-1, followed by kidney ischemia for 45 minutes and reperfusion for 24 hours. (a) Kidney lysates from WT mice after medical preconditioning were subjected to enzyme assay for ALDH2. *n* = 3. (b) Kidney lysates from WT mice were subjected to western blot analysis for ALDH2. *n* = 3/group. (c) Western blot analysis for ALDH2 protein in kidney samples from each group. *n* = 3/group. (d) Schematic diagram of Alda-1 and CYA preconditioning with ischemia–reperfusion (e) Serum creatinine (SCr) were measured after 24 h of reperfusion. *n* = 3. (f) H&E staining showed the injury of renal tubular epithelial cells and accumulation of red blood cells. Scale bar: 200 *μ*m, 50 *μ*m. (g) Paller score was performed using a semiquantitative damage assessment of renal tubular epithelial cells for each sample. *n* = 3. (h) Representative images of TUNEL staining (green) and DAPI (blue). Scale bar: 100 *μ*m. (i) The percentage of TUNEL staining-positive cells in the total cells in the random field. *n* = 3/group. (j) Morphology of renal tubular epithelial cell was observed under TEM. *n* = 3/group. Scale bar: 20 *μ*m, 10 *μ*m. Data are presented as the mean ± SD. ^∗^*P* < 0.05, ^∗∗^*P* < 0.01, and ^∗∗∗^*P* < 0.001 by *t*-test.

Our previous results showed that when ALDH2 is knocked out, kidney resistance to IRI is reduced, and the inflammatory response after IRI may be promoted through the phosphorylation of p65 or high expression of IL-17C. However, in the preliminary experiments, we found that ALDH2 activity only changed in animals and humans, while the protein expression level did not change. KO could not completely simulate the ALDH2 expression phenotypes under normal conditions. We used drugs targeting ALDH2 to illustrate the importance of ALDH2. We pretreated WT mice with agonists and inhibitors of ALDH2 (Figure 5(d)); the activity of ALDH2 was increased to approximately 1.5 times its original value with Alda-1 treatment, while it was decreased to 1/4 of its original value with CYA treatment (Figure S1D). There were no significant changes in kidney function or the pathological scores of the mice (Figure S1A-C). The results showed that the expression of ALDH2 in mouse kidney did not change significantly during IR, and the degree of kidney injury was related to the activity of ALDH2 (Figures 5(a)–5(c)). The creatinine level (Figure 5(e)), pathological degree of kidney tissue injury (Figures 5(f) and 5(g)), and apoptosis level (Figures 5(h) and 5(i)) in the Alda-1+IR group were lower than those in the NC+IR group. In contrast, ALDH2 inhibition worsened injury. The TEM results showed that compared to those in the NC+IR group, the mitochondria in the Alda-1+IR group showed significant improvements in condition (Figure 5(j)). Additionally, in kidney tubular epithelial cells, there were more mitochondria in the Alda-1+NC group than in the Alda-1+IR group; these mitochondria were similar to normal mitochondria and slightly enlarged, but the main mitochondrial structure was not damaged. In the corresponding CYA+IR group, the degree of mitochondrial damage was similar to that in the KO-IR group, with swelling and rupture, and the mitochondrial structure basically disappeared. It has been shown that ALDH2 can affect the morphology of mitochondria in IRI and strengthen mitochondrial tolerance to IR in kidney tubular epithelial cells. Finally, PCR was used to examine the increased activity of the inflammatory cytokines IL-10*β*, TNF-*α*, and MCP-1 (Figures 4(a)–4(c)) and that of ALDH2 in kidney tissues and to examine the reduced mRNA expression levels of these inflammatory cytokines.

### 3.5. ALDH2 Is Upstream of I*κ*B*α*/NF-*κ*B, and ALDH2 Regulation Affects the Phosphorylation of I*κ*B*α*/NF-*κ*B

In previous experiments, we demonstrated that the phosphorylation of I*κ*B*α*/NF-*κ*B was significantly increased after ALDH2 KO^−^. To further explore the relationship between ALDH2 and I*κ*B*α*/NF-*κ*B, western blot analysis was performed on NC+IR, CYA+IR, and Alda-1+IR group (Figure 4(d)). We found that I*κ*B*α* phosphorylation was regulated by ALDH2. p-I*κ*B*α*/I*κ*B*α*, p-p65/p65, and IL-17C levels were significantly decreased by ALDH2 activation; their levels were higher in the NC+IR group than in the CYA+IR group and lower in the NC+IR group (Figures 4(e)–4(g)). These results suggest that ALDH2 may be upstream of p-I*κ*B*α*/I*κ*B*α*, p-p65/p65, and IL-17C, which may further regulate renal IRI by affecting the expression of these molecules.

### 3.6. In HK-2 Cells, Exacerbation of IRI Caused by ALDH2 Inhibition Was Abrogated by NF-*κ*B Inhibitors

**Figure 6:**
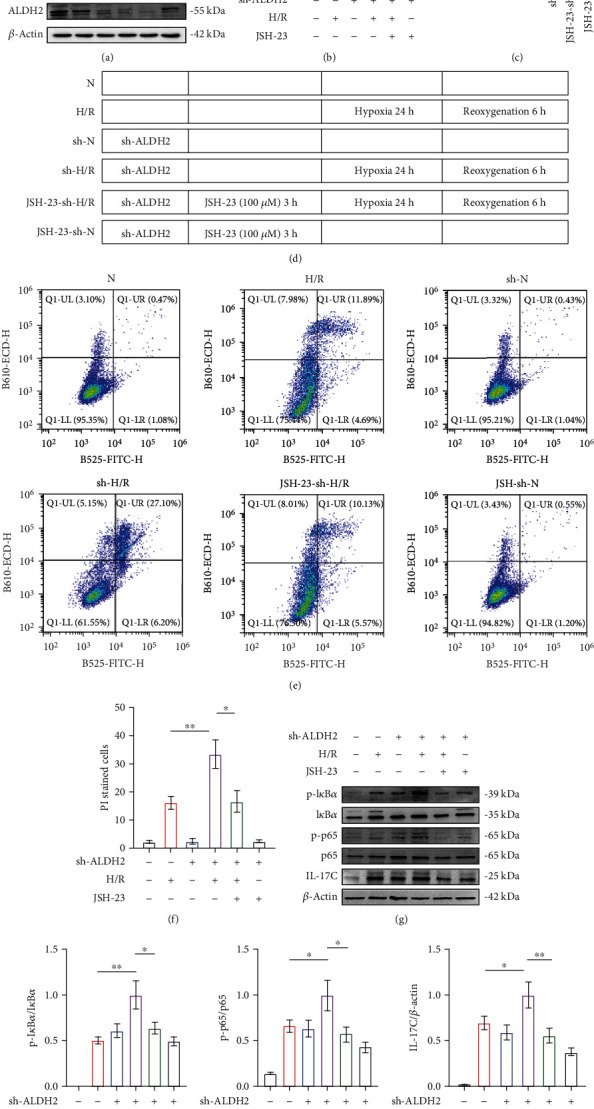
NF-*κ*B phosphorylase inhibitors attenuate the aggravation of injury caused by ALDH2 deficiency. (a) The western blot analysis of ALDH2 in HK-2 lysates. *β*-Actin was the standard. *n* = 3/group. (b) Kidney lysates from WT mice were subjected to western blot analysis for ALDH2. *n* = 3/group. (c) Cell lysates from HK-2 cell after knockdown ALDH2 and JSH-23 preconditioning were subjected to enzyme assay for ALDH2. *n* = 3/group. (d) Schematic diagram of HK-2 cell treatment. (e) The apoptosis of HK-2 cells was measured by flow cytometry, and the statistical results were expressed by histogram (f). *n* = 3/group. (g) The western blot band of p-I*κ*B*α*, I*κ*B*α*, p-p65, p65, and IL-17C in kidney lysates. *β*-Actin was the standard. *n* = 3/group. (h)–(j) Western blot analysis for p-I*κ*B*α*, I*κ*B*α*, p-p65, p65, and IL-17C protein in HK-2 cell samples from each group. *n* = 3/group. Data are presented as the mean ± SD. n.s.: not significant. ^∗^*P* < 0.05, ^∗∗^*P* < 0.01, and ^∗∗∗^*P* < 0.001 by *t*-test.

To verify our hypothesis, we established stable ALDH2 interference cell lines using HK-2 cells and verified them by western blotting. We used the p65 inhibitor JSH-23 to inhibit p65 phosphorylation but did not affect the expression of normal p65 (Figure 6(d)). At the same time, we determined the expression and activity of ALDH2 in each group (Figures 6(a)–6(c)). Pretreatment with JSH-23 for 3 hours, hypoxia for 24 hours, and reoxygenation for 6 hours were used on the sh group. Based on flow cytometry, sh-N cells were more likely to undergo apoptosis after H/R than normal HK-2 cells. However, when JSH-23 pretreatment was performed on sh-HK2 cells, the apoptosis level returned to a level similar to that in the HK2+H/R group, suggesting that inhibiting P65 phosphorylation could alleviate the adverse effects of ALDH2 inhibition (Figures 6(e) and 6(f)). To verify this hypothesis, we performed immunoblotting analysis of the cells and found that the expression of p-I*κ*B*α*/I*κ*B*α* was still higher than normal in the JSH-23-si-H/R group, but the expression of p-p65 and IL-17C was decreased. These results suggest that ALDH2 can affect IL-17C expression by regulating the phosphorylation of I*κ*B*α*/NF-*κ*B (Figures 6(g)–6(j)).

## 4. Discussion

In the past few decades, research on renal ischemia–reperfusion has included apoptosis, necrosis, autophagy, pyroptosis, ferroptosis, inflammation, and other aspects [28–31]. Multiple death modes jointly lead to injury to renal tissue. ALDH2 can reduce the extent of renal IRI, but the underlying mechanism remains unclear. Our results suggest that ALDH2 reduces inflammation by inhibiting the I*κ*B*α*/NF-*κ*B pathway during renal IRI.

In our previous study, we demonstrated that activation of ALDH2 alleviated renal edema and IRI in a cryogenic mechanical perfusion model in New Zealand rabbits; however, the underlying mechanism has not been well explored. Therefore, in this study, we further confirmed the important role of ALDH2 in renal IRI and further explored the possible molecular mechanism of ALDH2 in IRI.

ALDH2 is a member of the aldehyde dehydrogenase family; its family member ALDH1 has attracted attention for its role in the treatment of carcinogenesis and cancer, while ALDH2 has been studied for its promotion of alcohol metabolism and protection against alcoholism. However, in recent years, the role of ALDH2 in the pathogenesis and treatment of some diseases has attracted attention. The main function of ALDH2 is to remove endogenous aldehydes accumulated by lipid peroxidation in the process of oxidative stress; these aldehyde include 4-hydroxy-2-nonaldehyde (4-HNE) and malondialdehyde [32]; many diseases exhibit resistance to oxidative stress by mechanisms such as the consumption of 4-HNE. Early studies mostly suggested that the protective effect of ALDH2 on organ IRI was only based on the detoxification effect of the enzyme, which could affect autophagy, apoptosis, and other death modes to protect cells. However, recent studies have shown that ALDH2 interacts with other molecules that regulate cell biological activities in various models to produce a series of protective effects. Xu et al.'s study showed that activation of ALDH2 can promote Beclin-1 expression and destroy the interaction between Beclin-1 and Bcl-2, promoting autophagy [12]. Our team's early experiments also found that activation of ALDH2 can activate autophagy in the renal IRI process [13].

To explore the effect of ALDH2 deficiency on IRI, genome-wide transcript data from ALDH2^−/−^ and WT mouse renal cortex samples were assessed. According to RNA-Seq results, ALDH2 knockout leads to upregulation of some genes in the NF-*κ*B and IL-17C pathways during renal ischemia–reperfusion. Based on our results, ALDH2 deletion can promote the phosphorylation of I*κ*B*α*/NF-*κ*B p65 and the expression of IL-17C. These findings suggest potential new associations for ALDH2 and NF-*κ*B pathway-mediated inflammatory responses in renal IRI models.

NF-*κ*B is a member of the transcription factor family, widely exists in all mammals, and is believed to be involved in inflammation, apoptosis, autophagy, and other biological activities. In the process of ischemia–reperfusion, a variety of stimuli induce the degradation of I*κ*B and release of the NF-*κ*B p50-p65 dimer to activate NF-*κ*B signal transduction [33, 34]. Currently, it is believed that after the activation of the NF-*κ*B signaling pathway, the transcription of inflammatory factors such as TNF-*α* and IL-6 increases, leading to the activation of inflammatory factors and proteasomes, leading to further tissue damage. You et al. [35] used PYR-41 to inhibit the stimulation of lipopolysaccharide on macrophages and inhibit the expression of TNF-*α*, thus inhibiting the activation of NF-*κ*B and reducing the expression of intestinal proinflammatory cytokines after IRI. Li et al. [36] used MG132 to inhibit the NF-*κ*B pathway and reduce the extent of liver injury. Some natural antioxidants can also protect against IRI through their own antioxidant effects and inhibition of the NF-*κ*B pathway [37]. Ischemic preconditioning can also reduce the activation of the NF-*κ*B pathway during the subsequent IR process to some extent, resulting in a protective effect on organs. In our study, ALDH2 deficiency led to phosphorylation activation of I*κ*B*α*/NF-*κ*B p65 and simultaneously increased renal IRI in mice. We activated ALDH2 to reduce the phosphorylation of related molecules and reduce the extent of renal IRI, which is consistent with current studies on the NF-*κ*B pathway in IR. In in vitro experiments, we found that inhibiting the phosphorylation of NF-*κ*B can attenuate the damaging effect of ALDH2 inhibition on H/R models of cells, demonstrating that the NF-*κ*B pathway is an important pathway by which ALDH2 affects biological activities.

Interestingly, we also found that the IL-17 pathway was significantly altered by ALDH2 KO according to the RNA-Seq results and that the expression of IL-17C increased with the decrease in ALDH2 expression or activity.

IL-17A was characterized in 1993, and the interleukin-17 family has gradually expanded from to include six similar but different families, A, B, C, D, E, and F [38]. The specific role of IL-17A and IL-17C in IRI has been studied in mouse models, where early production of IL-23 and IL-12 after renal IRI activates the downstream IL-17A and IFN-*γ* signaling pathways and promotes renal inflammation [22]. Application of neutralizing monoclonal anti-IL-17A antibody resulted in reduced renal IRI, decreased renal and plasma concentrations of pro-inflammatory mediators, and increased renal and plasma levels of anti-inflammatory cytokines. Yang et al. [39] also found that IL-17A promotes neutrophil infiltration and mitochondria-driven apoptosis in fatty liver. Hepatic ischemia–reperfusion injury aggravates fatty liver. However, in the study of Thorenz et al. [40], IL-17A did not play a decisive role in severe renal ischemia–reperfusion injury, and the use of neutralizing monoclonal antibody could not effectively reduce the inflammation and later renal fibrosis caused by renal IRI. In another experiment, Wang et al. [22] found that IL-17C may be involved in the inflammatory response of acute kidney injury. Inhibition of IL-17C or blocking of IL-17RE can alleviate renal IRI, but the specific mechanism remains unclear. We demonstrate here that ALDH2 can reduce IL-17C expression by reducing the phosphorylation of I*κ*B*α*/NF-*κ*B p65, but the role of IL-17 family members in ischemia–reperfusion remains to be explored.

In summary, we demonstrated that ALDH2 can protect against IRI by reducing the phosphorylation of I*κ*B*α*, reducing the phosphorylation of p65, activating nucleation, and reducing the expression of downstream inflammatory factors.

## 5. Conclusions

**Figure 7:**
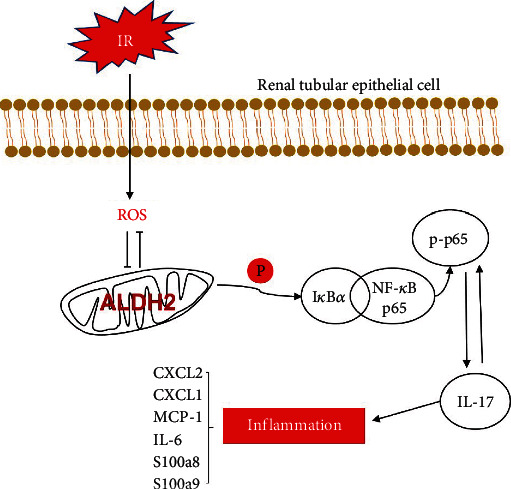
Molecular representation of ALDH2 after renal ischemia–reperfusion. After IR, renal tubular epithelial cells inhibit the activity of ALDH2 through oxidative stress, thereby promoting the phosphorylation of I*κ*B*α*, leading to the increased phosphorylation of NF-*κ*B and its entry into the nucleus, leading to the increased expression of various inflammatory factors including IL-17C, and finally leading to cell death.

In conclusion, this study found that in the process of renal ischemia–reperfusion injury, due to oxidative stress and other stimuli, ALDH2 deficiency can lead to the increase of mRNA and protein expression of I*κ*B*α*, stimulate phosphorylation of I*κ*B*α*, make NF-*κ*B phosphorylation into the nucleus, and further increase the expression of IL-17C and other inflammatory factors, leading to apoptosis (Figure 7). It aggravated the ischemia–reperfusion injury. Our inhibition of NF-*κ*B phosphorylation terminated the effects of ALDH2 deficiency. This may provide new ideas for the treatment of ALDH2-deficient transplant patients in the future.

## Data Availability

Data for the current study are available from the appropriate authors upon reasonable request.

## Supplementary Materials

Supplementary Materials Table S1: primers used for RT-PCR. Figure S1: ALDH2 activation or inhibition does not affect normal kidney morphology and function. WT mice were treated with drugs only. (A) H&E staining showed renal tubule epithelial cell injury and red blood cell deposition. Scale bar: 200 *μ*m, 50 *μ*m. *n* = 3/group. (B) Paller score was performed using a semiquantitative damage assessment of renal tubular epithelial cells for each sample. *n* = 3/group. (c) Serum creatinine (SCr) were measured after the medication is processed. *n* = 3/group. (d) Kidney lysates from WT mice after medical preconditioning were subjected to enzyme assay for ALDH2. *n* = 3.
