# Supplementary material for: Aldehyde Dehydrogenase 2 Protects the Kidney from Ischemia–Reperfusion Injury by Suppressing the IκBα/NF-κB/IL-17C Pathway
Source: Oxid Med Cell Longev. 2023 Feb 21;2023:2264030. doi: 10.1155/2023/2264030 (PMC9974261; doi:10.1155/2023/2264030)
Supplement: Supplementary Materials — Table S1: primers used for RT-PCR. Figure S1: ALDH2 activation or inhibition does not affect normal kidney morphology and function. WT mice were treated with drugs only. (A) H&E staining showed renal tubule epithelial cell injury and red blood cell deposition. Scale bar: 200 μm, 50 μm. n = 3/group. (B) Paller score was performed using a semiquantitative damage assessment of renal tubular epithelial cells for each sample. n = 3/group. (c) Serum creatinine (SCr) were measured after the medication is processed. n = 3/group. (d) Kidney lysates from WT mice after medical preconditioning were subjected to enzyme assay for ALDH2. n = 3. [file 2264030.f1.docx]

Supplemental Files

Aldehyde dehydrogenase 2 protects the renal from ischemia reperfusion injury by suppressing the IκBα/NF-κB/IL-17C pathway

Yiwen Chen^1^, Yan Xiong^1^, Jun Luo^1^, Qianchao Hu^1^, Jianan Lan^1^, Yongkang Zou^1^, Qin Ma^1^, Hanlin Yao^1^, Zhongzhong Liu^#1^, Zibiao Zhong^#1^ and Qifa Ye^#1,2^

1. Zhongnan Hospital of Wuhan University, Institute of Hepatobiliary Diseases of Wuhan University, Transplant Center of Wuhan University, National Quality Control Center for Donated Organ Procurement, Hubei Key Laboratory of Medical Technology on Transplantation，Hubei Clinical Research Center for Natural Polymer Biological Liver, Hubei Engineering Center of Natural Polymer-based Medical Materials, Wuhan Hubei，430071；
2. The 3rd Xiangya Hospital of Central South University, Research Center of National Health Ministry on Transplantation Medicine Engineering and Technology, Changsha, 410013.

Corresponding author. Qifa Ye: yqf_china@163.com

Zibiao Zhong: [hizzb@whu.edu.cn](mailto:hizzb@whu.edu.cn)

Liu Zhongzhong: liuzhongzhong28@163.com

# Corresponding author

Table S1

Figure S1

Table S1. Primers used for RT-PCR.

| **Genes** | **Forward** | **Reverse** |
| --- | --- | --- |
| GAPDH | TCGCTCCTGGAAGATGGTGAT | CAGTGGCAAAGTGGAGATTGTTG |
| ALDH2 | AACAATGAGTGGCACGACG | TGCATCAGGAGCGGGAAG |
| HSP90 | GGAGCTGCACATCAATCT | ATCGTCGTTATGCTTCGT |
| JUN | AGCGTGTTCTGGCTATGC | CGGACCGTTCTATGACTGC |
| IκBα | TTTGCCACTTTCCACTTAT | AATCCTGACCTGGTTTCG |
| Gldc | AGGGAAGGAAGTGTATCGC | AGATGACTCACAGCCGAAG |
| S100a9 | TACTGGGCTTACACTGCT | CATTCCCTTTAGACTTGG |
| IL-17A | TGTGAAGGTCAACCTCAAAGTCT | GAGGGATATCTATCAGGGTCTTCAT |
| IL-17B | GAGTATGAGCGGAACCTTGG | CTGGGGTCGTGGTTGATG |
| IL-17C | CCTCTAGCTGGAACACAGTGC | GCGGTTCTCATCTGTGTCG |
| IL-17D | TCCGGCCACCCACCAACCTG | ACAGGCAGTAGGCTTCGGGCAGGTA |
| IL-17E | TGGAGCTCTGCATCTGTGTC | GATTCAAGTCCCTGTCCAACTC |
| IL-17F | GGACTTGCCATTCTGAGGGAGGTAGC | CCGGTGGGGGTCTCGAGTGATGT |
| IL-10 | CTTACTGACTGGCATGAGGATCA | GCAGCTCTAGGAGCATGTGG |
| MCP-1 | TAAAAACCTGGATCGGAACCAAA | GCATTAGCTTCAGATTTACGGGT |
| TNF-α | CAGGCGGTGCCTATGTCTC | CGATCACCCCGAAGTTCAGTAG |


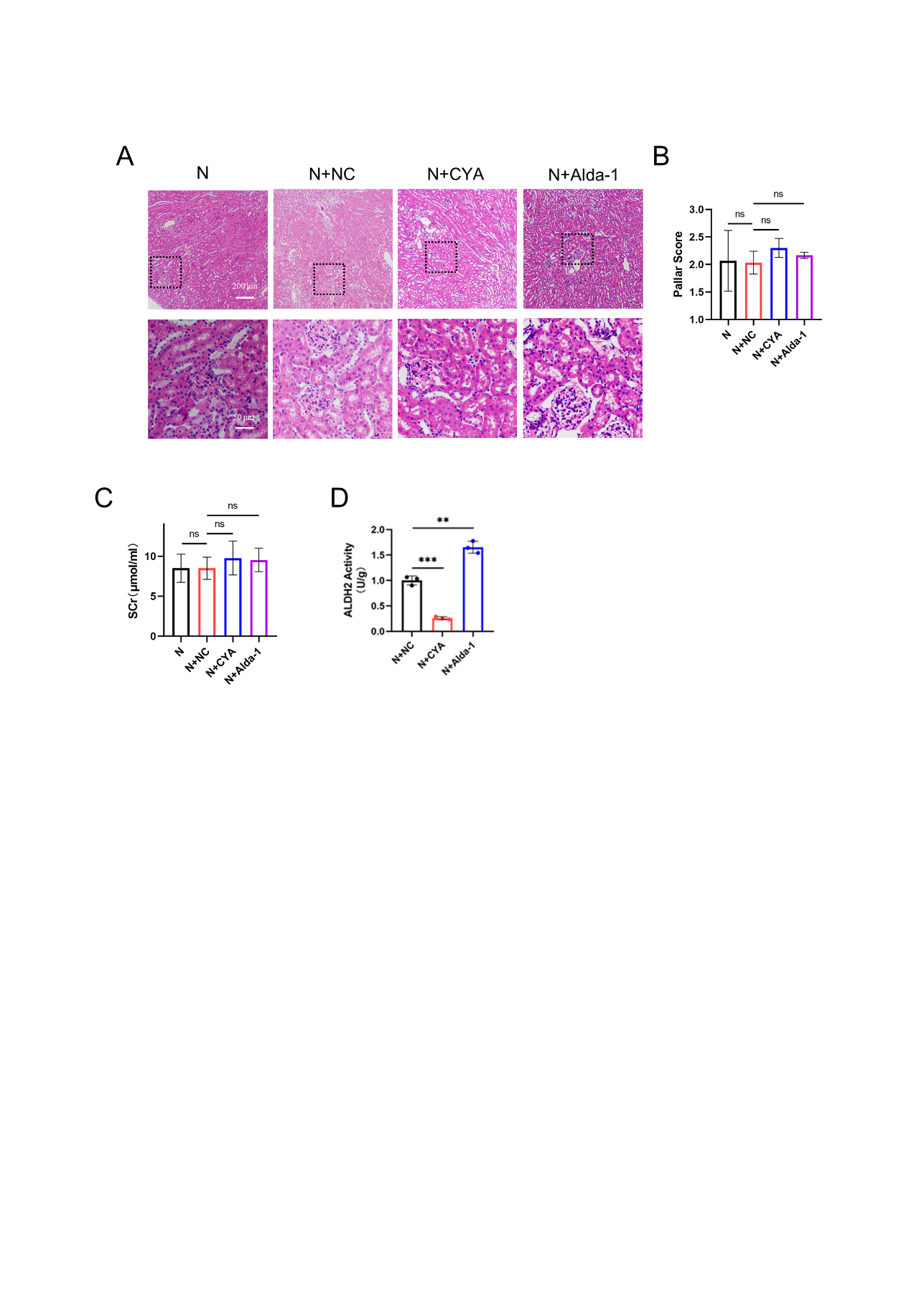


Figure S1：ALDH2 activation or inhibition does not affect normal kidney morphology and function

WT mice were were treated with drugs only. (A) H&E staining showed Renal tubule epithelial cell injury and red blood cell deposition. Scar bar: 200 μm, 50 μm. n = 3/group. (B) Paller score was performed using a Semi-quantitative damage assessment of renal tubular epithelial cells for each sample. n = 3/group. (C) Serum creatinine (SCr) were measured after the medication is processed. n = 3/group. (D) Kidney lysates from WT mice after medical preconditioning were subjected to enzyme assay for ALDH2. n = 3.
